# Volatile Profiling and Transcriptomic Analysis of Peel and Flesh in Wampee (*Clausena lansium* (Lour.) Skeels)

**DOI:** 10.3390/metabo16080598

**Published:** 2026-08-21

**Authors:** Ruibing Xu, Qingshan Li, Gengrui Zhu, Yi Chen, Gaoyang Zhang

**Affiliations:** 1 Key Laboratory Forest Tree Genetics & Breeding of Liaoning Province, College of Forestry, Shenyang Agricultural University, Shenyang 110866, China; ruibing_ice@163.com; 2 Guangxi Key Laboratory of Agro-Environment and Agro-Products Safety, National Demonstration Center for Experimental Plant Science Education, College of Agriculture, Guangxi University, Nanning 530004, China; sichenff@163.com (Q.L.);; 3 College of Life Sciences, Shangrao Normal University, Shangrao 334001, China; 4 Institute of Precision Medicine, Zhejiang Chinese Medical University, Hangzhou 311400, China

**Keywords:** wampee, transcriptomic, peel, flesh, VOCs

## Abstract

**Background**: Wampee (*Clausena lansium* (Lour.) Skeels) is an understudied Rutaceae crop native to southern China, whose fruit features a complex aroma profile with simultaneous sour, sweet, bitter and astringent notes. Although bioactive compounds including flavonoids, alkaloids and volatile oils in wampee fruit have been partially characterized, the tissue-specific metabolic and transcriptional basis underlying its distinctive aroma formation remains largely unclear. **Methods**: We performed an integrated volatile metabolomic and transcriptomic analysis on the pericarp (peel) and flesh of wampee fruit across three cultivars (Shanyellowpi, Heijingang, Bingtangxin). Volatile metabolites were profiled via headspace solid-phase microextraction coupled with gas chromatography-mass spectrometry (HS-SPME-GC-MS), and transcriptome profiles were generated by RNA-Seq. Multi-omics integration was conducted using Procrustes analysis, gene–metabolite correlation network construction and weighted gene co-expression network analysis (WGCNA). **Results**: A total of 288 volatile metabolites were identified, representing the most comprehensive volatile inventory for *C. lansium* reported to date. Principal component analysis and partial least squares discriminant analysis revealed distinct volatile profiles between pericarp and flesh; terpenoids were the dominant chemical class, accounting for 71.56–91.48% of total volatiles in pericarp and 61.88–67.22% in flesh. Notably, organoheterocyclic compounds were significantly enriched in Shanyellowpi flesh (40.45%), forming a cultivar-specific metabolic signature absent in the other two cultivars. Transcriptomic analysis showed that phenylpropanoid biosynthesis was the most significantly enriched pathway among differentially expressed genes, followed by monoterpene biosynthesis and sesquiterpenoid biosynthesis. Procrustes analysis demonstrated a strong global concordance between the two omics layers (M^2^ = 0.535, *p* < 0.001). Gene–metabolite correlation networks identified terpene synthase (*TPS*) genes (*HP075360*, *HP217350*) and oxidoreductase genes (*SOD1*, *GST*, *10HGO*) as candidate co-regulators of terpenoid biosynthesis. WGCNA further prioritized *TPS* genes, cytochrome P450 genes and MYB transcription factor genes as key regulators driving volatile metabolic divergence between tissues. **Conclusion**: This study provides a comprehensive volatile and transcriptomic atlas of wampee fruit, and identifies tissue-specific and cultivar-specific metabolic signatures as well as their candidate regulatory genes. These findings advance our understanding of quality differentiation in Rutaceae fruits and lay a foundation for molecular breeding and flavor improvement of wampee.

## 1. Introduction

Wampee (*Clausena lansium* (Lour.) Skeels), a perennial evergreen tree in the Rutaceae family, is native to southern China and has long been cultivated for its edible fruit. The fruit is valued for its complex aroma profile—simultaneously sour, sweet, bitter, and astringent—and for its rich content of bioactive compounds, including flavonoids, alkaloids, and volatile oils, earning it the local designation “treasure among fruits” [1]. Recent studies have systematically characterized the chemical composition and tissue distribution of wampee fruit. Non-targeted metabolomics has revealed substantial differences in metabolite profiles between the pericarp and the flesh [2]. Tissue-specific metabolic analyses across cultivars have further elucidated how secondary metabolites differentiate between fruit compartments [3]. Targeted metabolomics combined with quantitative analysis has enabled the precise characterization of key phenolic compounds in the pericarp and flesh [4,5]. These tissue-specific differences in phenolic composition are directly relevant to both bioactivities—such as antioxidant and anti-inflammatory effects—and sensory attributes, including astringency and bitterness [3]. Proanthocyanidins in the flesh and characteristic alkaloids extracted from the whole fruit have all been shown to possess diverse pharmacological activities, providing a scientific basis for the deeper utilization of wampee [6].

Flavor, and particularly aroma, is a central quality trait in fruit. The distinctive “medicinal” and “citrus-like” notes of wampee are contributed by a complex blend of volatile organic compounds (VOCs). However, the pericarp and flesh—tissues that diverge substantially during fruit development—differ markedly in both the composition and the accumulation mechanisms of their volatile constituents. Elucidating these differences at both the metabolic and transcriptomic levels is essential for understanding the molecular regulation of flavor formation in wampee and for informing cultivation and breeding strategies.

VOCs in Rutaceae fruits are predominantly composed of monoterpenes and sesquiterpenes, a compositional signature that has been consistently documented across Citrus species [7]. In wampee, the pericarp contains a high concentration of aromatic oils, and its volatile constituents generally exceed those of the flesh in both abundance and diversity [4]. Beyond their role in aroma, these compounds serve as a physiological barrier against environmental stress, pathogens, and herbivores [7]. By contrast, although the flesh contains a diverse array of VOCs, their concentrations are comparatively low and are subject to tight transcriptional regulation during fruit ripening. Such tissue-specific partitioning of volatiles is not unique to wampee; it has been reported in banana, mango, and other fleshy fruits, suggesting a general principle of tissue functional differentiation during fruit development [8,9,10]. Nevertheless, comparative studies of aroma composition in wampee pericarp and flesh have largely remained at the metabolomic level, and the lack of integrated transcriptomic analysis has left our understanding of the spatiotemporal expression patterns of aroma-related genes—such as terpene synthases (TPSs) and lipoxygenases (LOXs)—incomplete.

Transcriptomics has become an indispensable tool for dissecting fruit quality formation, particularly following the completion of the wampee genome [5,6], which provides a critical genomic foundation for identifying key genes involved in aroma biosynthesis. Tissue-specific transcriptome profiling enables the construction of detailed gene expression maps and the identification of regulators governing metabolic pathways. During fruit development and ripening, transcription factors—notably MYB, WRKY, and bHLH family members—play central roles in modulating aroma biosynthesis pathways. Recent integrated transcriptomic and metabolomic analyses of wampee pericarp pigmentation and metabolite accumulation have identified key structural genes and transcription factors underlying these quality traits, establishing a methodological framework for multi-omics investigation of aroma [11,12]. Weighted gene co-expression network analysis (WGCNA) can further link metabolic and transcriptional data to identify candidate genes controlling tissue-specific volatile accumulation. This “metabolite-transcript” integrative strategy has been successfully applied to quality improvement in citrus and other fruits [13,14], and provides a robust theoretical framework for the present study.

Here we report an integrated volatile metabolomic and transcriptomic analysis of wampee pericarp and flesh. We found that terpenoids dominate the volatile profile and identified candidate TPS and MYB genes associated with tissue-specific aroma formation. Our findings advance the understanding of quality differentiation in Rutaceae fruits and provide a scientific basis for molecular marker-assisted breeding and targeted flavor modulation in wampee.

## 2. Materials and Methods

### 2.1. Plant Materials

Mature wampee fruits, *Clausena lansium* (Lour.) Skeels, were harvested on 1 September 2025 from the germplasm collection at the Fruit Tree Orchard, College of Agriculture, Guangxi University (108°17′42.37″ E, 22°50′28.98″ N). Three cultivars were collected (Table 1), with 30 uniform, undamaged, and disease-free fruits selected for each cultivar. Fruits were transported to the laboratory within 2 h of harvest. For each cultivar, the 30 fruits were allocated to 3 biological replicates of 10 fruits each; peel and flesh from each replicate were pooled and processed independently, yielding 3 independent biological replicates per tissue–cultivar combination for both GC–MS and RNA-Seq. The peel (P) and flesh (F) tissues were immediately separated using a sterile scalpel, frozen in liquid nitrogen, and stored at −80 °C until subsequent analysis.

### 2.2. Volatile Extraction and HS-SPME-GC–MS Analysis

Volatile compounds were extracted by headspace solid-phase microextraction (HS-SPME) and analysed by gas chromatography–mass spectrometry (GC–MS) [15]. Approximately 1.0 g of frozen tissue powder was placed in a 20 mL headspace vial containing saturated NaCl solution to minimise volatile solubility. 2-Octanol (1 µL, internal standard) was added to correct for extraction efficiency and instrument response drift. Vials were equilibrated at 60 °C for 15 min, after which a 50/30 µm DVB/CAR/PDMS fibre was inserted and allowed to adsorb volatiles for 40 min. Analyses were performed on an Agilent 7890B-5977A GC-MS, Agilent Technologies, Santa Clara, CA, USA system equipped with a DB-5MS capillary column (30 m × 0.25 mm × 0.25 µm). Helium was used as the carrier gas at a constant flow rate of 1.0 mL min^−1^. The oven temperature programme was as follows: 40 °C (hold 3 min), ramped at 5 °C min^−1^ to 200 °C, then at 10 °C min^−1^ to 250 °C (hold 5 min). Electron ionisation (EI) was operated at 70 eV; the ion source and transfer line were maintained at 230 °C. Full-scan mode covered *m*/*z* 35–450. Compounds were identified by matching mass spectra against the NIST 21 mass spectral library and confirmed by retention index (RI) [16]. Relative quantities were calculated by the internal standard method. Retention indices were calculated from a series of n-alkanes (C7–C40) analysed under identical chromatographic conditions using the Kovats method. Absolute quantification using stable-isotope-labelled internal standards was not performed; reported values are relative abundances normalized to the 2-octanol internal standard [17,18]. Each biological replicate was analysed in 3 technical replicate(s). Volatiles were separated in splitless mode. Instrument control and raw-data processing were performed with Agilent MassHunter Qualitative Analysis B.07.00; Agilent Technologies, Santa Clara, CA, USA [19].

### 2.3. Transcriptome Sequencing and Bioinformatics Analysis

Total RNA was extracted from peel and flesh tissues using a modified Trizol method. RNA purity and concentration were assessed with a NanoDrop 2000 spectrophotometer; Thermo Fisher Scientific, Waltham, USA, and RNA integrity was verified using an Agilent 2100 system, with RNA integrity number (RIN) > 7.0. mRNA was enriched using Oligo (dT) magnetic beads, followed by random fragmentation, reverse transcription, end repair, 3′-adenylation, and adapter ligation for library construction. Paired-end sequencing (2 × 150 bp) was performed on an Illumina HiSeq X Ten platform. Sequencing generated an average of 5.52 Gb of clean data per sample, corresponding to an average sequencing depth of 20-fold.

Raw reads were processed by Fastp (v0.23.2) to remove adapter sequences, poly-N stretches, and low-quality bases to obtain high-quality clean reads. Clean reads were aligned to the wampee reference genome (*Clausena lansium* HPv1.0.genome, https://www.citrusgenomedb.org/Analysis/2898456, accessed on 23 June 2026) using HISAT2 (v2.2.1). Gene expression was quantified by FeatureCounts (v2.0.6) and normalized to transcripts per million (TPM). Differentially expressed genes (DEGs) between peel and flesh tissues were identified using DESeq2 (v1.40.2) implemented under R (v4.3.2), with the thresholds set as |log_2_FoldChange| > 1 and adjusted *p*-value < 0.05.

### 2.4. Integrated Metabolomic and Transcriptomic Analysis

To elucidate the molecular regulation of flavour formation in wampee fruit, a combined metabolomic–transcriptomic analysis was performed [20]. Differential volatile compounds (DVs) and DEGs were subjected to KEGG enrichment analysis to identify shared metabolic pathways (e.g., terpenoid biosynthesis, fatty acid metabolism) [21,22]. Pearson correlation analysis was used to link volatile concentrations with the expression levels of candidate biosynthesis genes (|r| > 0.8, *p* < 0.05). Differential volatile compounds (DVs) were defined by VIP > 1 and *p* < 0.05 from the PLS-DA model. Gene–metabolite Pearson correlations were considered significant at *p* < 0.05, and the Benjamini–Hochberg false-discovery rate (FDR) was used for multiple-testing correction with an adjusted *p*-value threshold of 0.05. Weighted gene co-expression network analysis (WGCNA, R package v1.72) was applied to extract gene modules highly correlated with key aroma compounds. A signed weighted co-expression network was built using a soft-thresholding power (β = 14) selected to satisfy a scale-free topology fit (R^2^ ≥ 0.80). Modules were identified by dynamic tree cutting (deepSplit = 2, minModuleSize = 30) and merged when eigengene correlations exceeded 0.75. Transcription factor prediction was integrated to construct a core regulatory network. All bioinformatics analyses and visualisations were performed in R, with supplementary visualisation via the Majorbio Cloud platform (www.majorbio.com, accessed on 27 June 2026) [23,24]. The 20 candidate genes included in the gene–metabolite correlation network were selected as the DEGs showing the strongest tissue-specific expression (|log_2_FC| > 4) and the highest Pearson correlation (|r| > 0.8, *p* < 0.05) with the top differential volatiles, prioritising known volatile-biosynthesis gene families (TPS, GST, SOD1, 10HGO, ADH1, AAT) [25,26,27,28].

### 2.5. Validation of Gene Expression by qRT-PCR

Ten candidate genes potentially involved in volatile biosynthesis were selected for qRT–PCR validation. Gene-specific primers were designed using Primer Premier 6.0 based on the wampee reference genome (Table 2). Primer specificity was verified by NCBI Primer-BLAST (https://www.ncbi.nlm.nih.gov/tools/primer-blast/, accessed on 29 June 2026).and agarose gel electrophoresis. Total RNA (1 µg) was reverse-transcribed using the PrimeScript™ RT Reagent Kit with gDNA Eraser (TaKaRa Bio Inc., Kusatsu, Japan). qRT-PCR was performed on a Bio-Rad CFX96 system using SYBR Green fluorescence. The 2^−ΔΔCt^ method was used to calculate relative expression, with Actin as the internal reference gene. qRT-PCR data were analysed with Bio-Rad CFX Manager v3.1 and LinRegPCR. Ten candidate genes were randomly chosen for validation. These genes were prominent peel- or flesh-enriched DEGs. All data are expressed as mean ± SD (*n* = 3), with each qRT-PCR reaction run in technical triplicate.

## 3. Results

### 3.1. Identification and Quantification of Volatile Compounds

A total of 288 volatile compounds were identified and classified into 17 chemical categories across the peel and flesh of three wampee cultivars using HS-SPME-GC–MS (Table S1). Terpenoids (83 compounds) were the predominant class, accounting for 71.56–91.48% in peels and 61.88–67.22% in flesh, serving as the primary source of characteristic wampee aroma. Organoheterocyclic compounds exhibited strong tissue specificity, reaching 40.45% in Shanyellowpi flesh (SF) but remaining below 5% in all other samples, suggesting their involvement in the unique aroma profile of this cultivar. Esters and alcohols consistently accumulated more in flesh than in peel, with alcohols peaking at 13.36% (TF) and 8.74% (ZF), likely reflecting precursor accumulation for ester biosynthesis. Notably, phenol esters were exclusively enriched in Heijingang peel (ZP, 1.34%), potentially as a cultivar-specific marker (Table 3).

**Figure 1:**
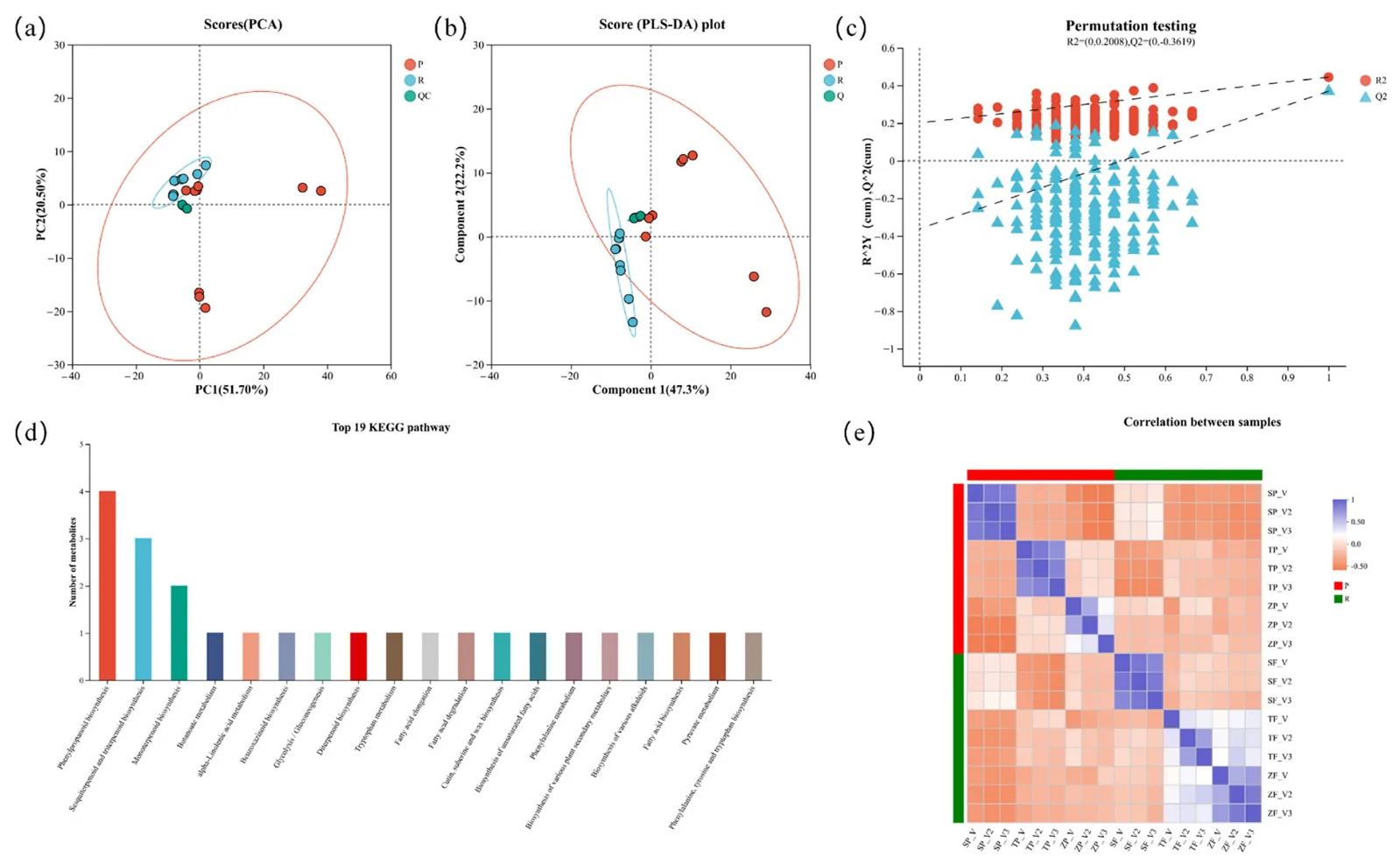
Multivariate statistical analysis and pathway enrichment of volatile metabolites in wampee Peel and flesh. (**a**) PCA score plot. (**b**) PLS-DA score plot. (**c**) Permutation testing for PLS-DA model. (**d**) KEGG pathway enrichment analysis of differential volatile metabolites. (**e**) Pearson correlation heatmap of volatile metabolite profiles across all biological replicates. Note: The dotted lines in panel (**c**) are regression lines of cumulative R^2^Y and Q^2^ derived from permutation testing, which assess over-fitting of the PLS-DA model. A negative intercept of the Q^2^ regression line indicates no over-fitting of the classification model.

Principal component analysis (PCA) revealed that PC1 and PC2 explained 72.20% of total variance (51.70% and 20.50%, respectively), with peel samples showing greater dispersion than flesh, indicating more pronounced inter-cultivar differences in peel tissues (Figure 1a). Quality control (QC) samples clustered tightly near the origin, confirming analytical stability. Partial least squares-discriminant analysis (PLS-DA) further demonstrated clear separation between peel and flesh along Component 1 (47.3%), with flesh forming a compact cluster while peel samples were more dispersed (Figure 1b). The partial least squares discriminant analysis (PLS-DA) was performed to systematically distinguish the metabolic differences between samples. The established PLS-DA model based on two principal components exhibited reliable explanatory and predictive capability. Specifically, the first principal component (p1) explained 0.473 of the total X-variance and 0.297 of the Y-variance, with a predictive Q^2^ value of 0.258. The second principal component (p2) further contributed 0.222 of X-variance and 0.147 of Y-variance, corresponding to a Q^2^ of 0.149 (Figure 1c). The cumulative explanatory parameters of the model reached R^2^X (cum) = 0.694 and R^2^Y (cum) = 0.444, and the cumulative predictive parameter Q^2^ (cum) was 0.369. To verify model robustness and eliminate overfitting bias, a 200-round permutation test was conducted. The permutation test yielded a negative Q^2^ intercept, which confirmed that the constructed PLS-DA model had no overfitting phenomenon and possessed stable and credible predictive performance for subsequent differential metabolite screening. KEGG enrichment analysis identified 19 significantly enriched pathways, with phenylpropanoid biosynthesis (4 metabolites), monoterpene biosynthesis (3), and sesquiterpenoid and triterpenoid biosynthesis (3) ranking highest (Figure 1d), highlighting terpenoid and phenylpropanoid metabolism as central to wampee aroma formation. Finally, Pearson correlation analysis confirmed high intra-group reproducibility (r > 0.90 within biological replicates), while peel–flesh anti-correlation patterns further supported tissue-specific metabolic reprogramming (Figure 1e).

**Figure 2:**
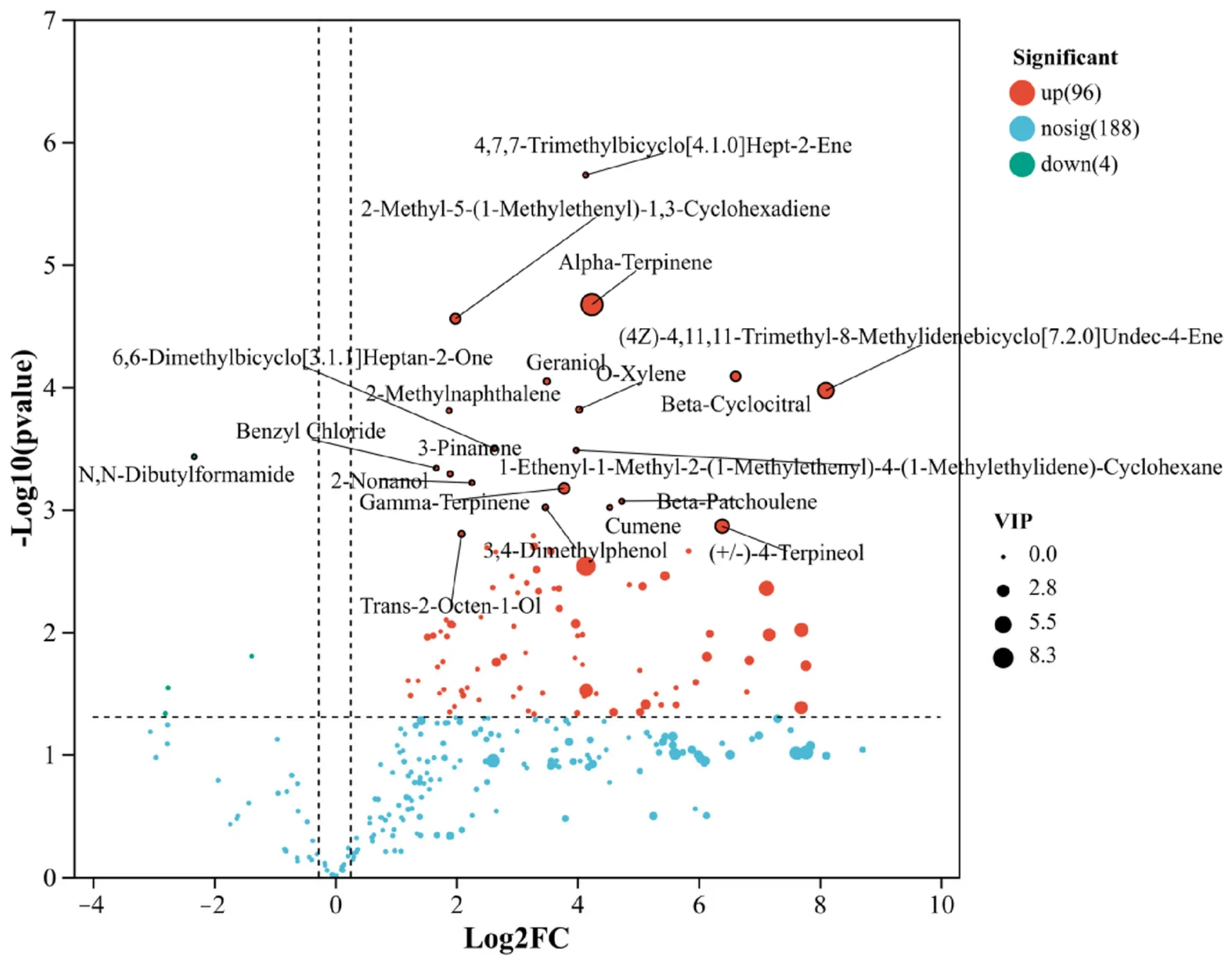
Differential volatile compounds between peel and flesh (volcano plot).

To identify key discriminant volatiles between peel and flesh tissues, a PLS-DA-based volcano plot was constructed combining variable importance in projection (VIP), fold change (FC), and statistical significance (Figure 2). Among 288 detected compounds, 96 were significantly up-regulated in flesh relative to peel (VIP > 1, *p* < 0.05), whereas only 4 were down-regulated, revealing an asymmetric accumulation pattern favoring flesh tissue. The top-ranked differential metabolites were predominantly terpenoids, including α-terpinene (Log_2_FC ≈ 4.2, VIP ≈ 5.5), γ-terpinene, and (±)-4-terpineol, alongside β-cyclocitral and o-xylene as notable non-terpenoid markers. Notably, 4,7,7-trimethylbicyclo[4.1.0]hept-2-ene exhibited the highest statistical significance, while (4Z)-4,11,11-trimethyl-8-methylidenebicyclo[7.2.0]undec-4-ene displayed the most extreme fold change (Log_2_FC ≈ 9). These results collectively indicate that terpenoid biosynthesis is the principal metabolic process associated with the distinct aroma profiles of wampee flesh versus peel.

Volcano plot of peel (P) vs. flesh (R) across three cultivars. Red and blue points denote significantly up- and down-regulated volatiles in flesh, respectively (*p* < 0.05, VIP > 1); gray points are non-significant. In total, 96 compounds were up-regulated and 4 were down-regulated in flesh. The top 20 differential volatiles ranked by VIP are labeled.

### 3.2. Quality Assessment of Transcriptome Sequencing

After filtering low-quality reads and adapter sequences using Fastp, an average of 40.80 million clean reads were obtained for each sample, with a Q30 percentage > 96.6% and GC content ranging from 43.55% to 44.58% (Table S2). Over 80% of clean reads were successfully mapped to the wampee reference genome, with uniquely mapped reads accounting for >74% of total reads.

### 3.3. Differential Gene Expression Between Peel and Flesh

**Figure 3:**
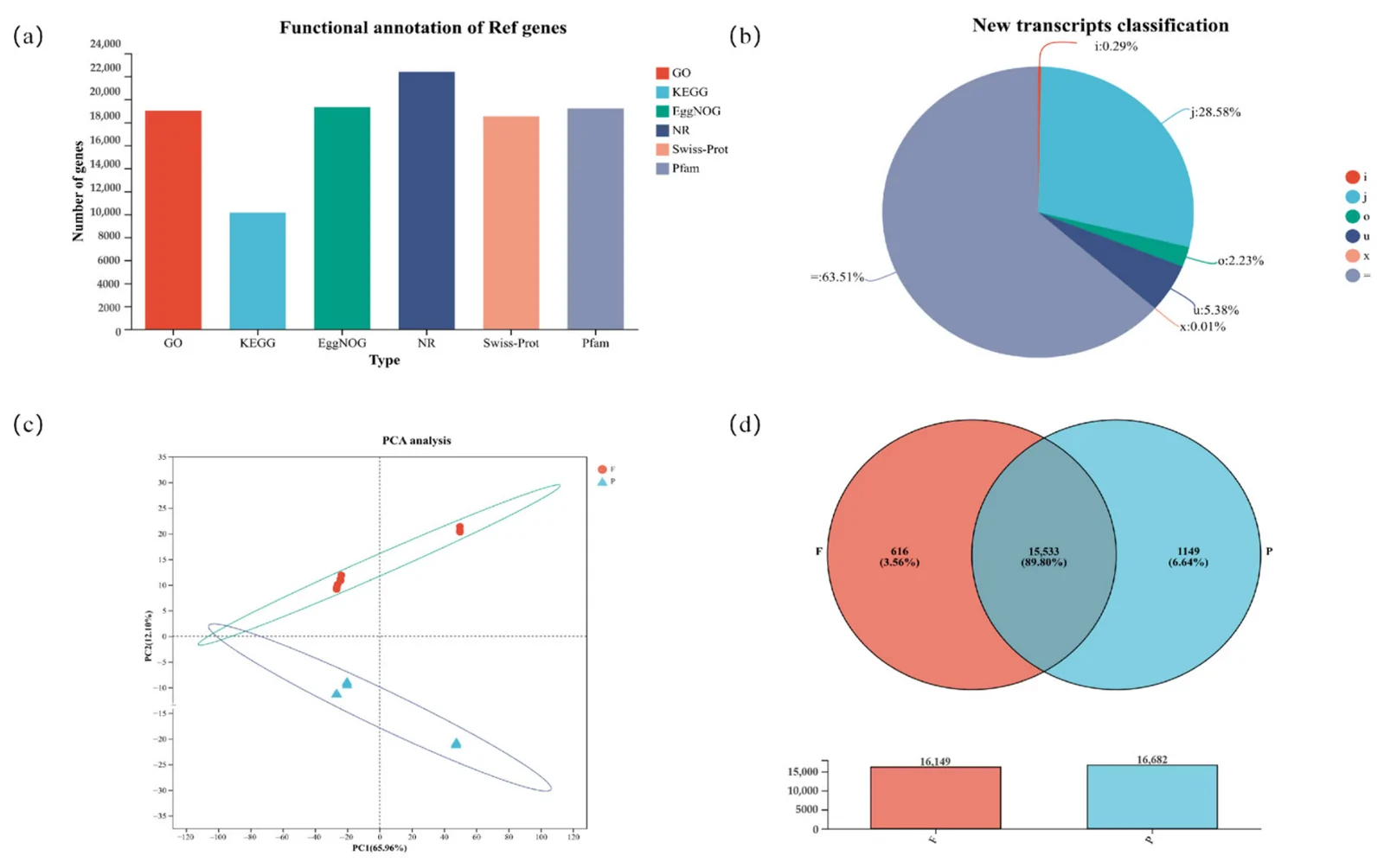
Transcriptome assembly, annotation, and comparative analysis of wampee fruit tissues. (**a**) Functional annotation of reference genes. Number of genes successfully annotated across six databases (GO, KEGG, EggNOG, NR, Swiss-Prot, Pfam). (**b**) Classification of newly assembled transcripts. Pie chart showing distribution of StringTie class codes. =, complete match of intron chain; i, potential novel isoform; u, unknown intergenic transcript; o, generic exonic overla (2.23%); j, transfrag within reference intron; x, antisense overlap. (**c**) PCA score plot of transcriptomic profiles. Red circles represent pericarp samples; blue triangles represent flesh samples. (**d**) Venn diagram of expressed transcripts.

A comprehensive transcriptome assembly was performed on RNA-Seq data from wampee peel and flesh tissues. Functional annotation against six major databases revealed that 22,497 reference genes were annotated in the NR database, followed by Pfam (18,717), EggNOG (19,016), GO (18,024), Swiss-Prot (17,826), and KEGG (9612) (Figure 3a). A total of 74,439 assembled transcripts were classified using StringTie, of which 63.51% (n = 47,277) exhibited complete intron chain matching with reference annotations (=), while 28.58% (n = 21,277) represented potentially novel isoforms (i) sharing at least one splice junction with known transcripts (Figure 3b). Intergenic transcripts (u, 5.38%, n = 4006) and transfrag-containing introns (j, 0.29%) accounted for minor fractions, indicating high assembly quality. PCA of gene expression profiles demonstrated clear separation between peel (P) and flesh (F) tissues along both principal components (Figure 3c), consistent with metabolomic findings. The Venn diagram further revealed that 15,553 transcripts (90.90%) were co-expressed in both tissues, while 616 and 1149 transcripts were uniquely detected in R and P groups, respectively (Figure 3d), suggesting tissue-specific transcriptional regulation underlying volatile metabolic divergence.

**Figure 4:**
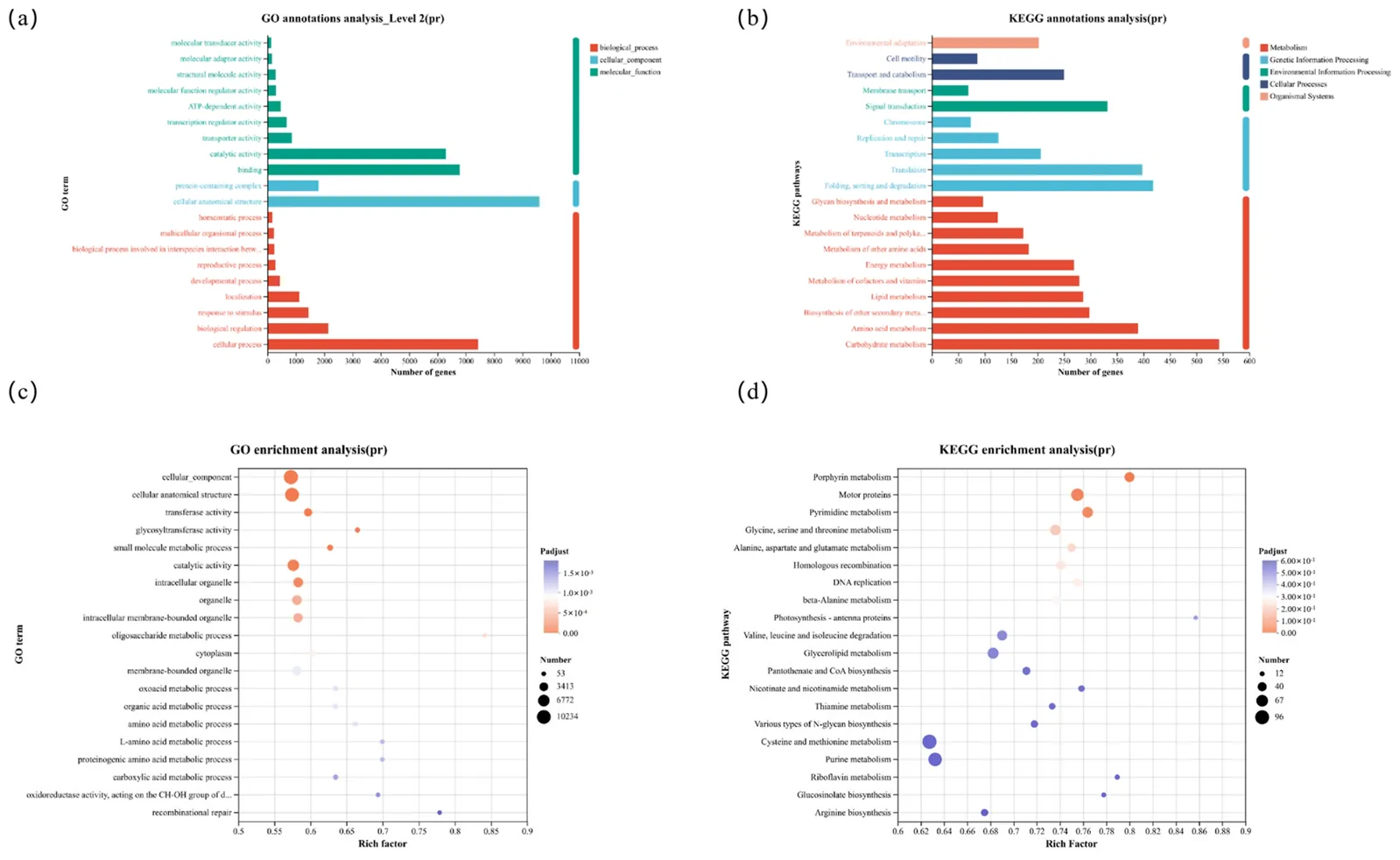
Functional annotation and enrichment analysis of wampee transcriptome. (**a**) GO functional annotation (Level 2). (**b**) KEGG pathway classification. (**c**) GO enrichment analysis of DEGs. (**d**) KEGG pathway enrichment analysis of DEGs. Differential expression analysis identified thousands of DEGs between peel and flesh, with up-regulated genes (red) substantially outnumbering down-regulated ones (blue).

To elucidate the molecular basis of volatile biosynthesis in wampee fruit, we performed an integrated analysis of DVs and DEGs. Gene Ontology (GO) annotation of the assembled transcripts revealed that cellular process and metabolic process dominated Biological Process (BP) terms, while catalytic activity and binding were the most abundant categories under Molecular Function (MF); cell and cell part represented the primary Cellular Component (CC) assignments (Figure 4a). KEGG pathway classification mapped ~22,497 genes into five major categories, with Metabolism (particularly carbohydrate metabolism, translation, and signal transduction) accounting for the largest proportion (Figure 4b). To elucidate transcriptional mechanisms underlying tissue-specific volatile metabolism, GO and KEGG enrichment analyses were performed on differentially expressed genes (DEGs). The top 20 significantly enriched GO terms (adjusted *p* < 0.05) were predominantly associated with cellular component organization, intracellular anatomical entity, membrane-bounded organelle, and oxidoreductase activity, indicating extensive structural and enzymatic reprogramming between tissues (Figure 4c). Notably, KEGG enrichment highlighted phenylpropanoid biosynthesis as the most significant pathway, followed by glyoxylate and dicarboxylate metabolism, alanine, aspartate and glutamate metabolism, and porphyrin metabolism (Figure 4d). These findings collectively suggest that phenylpropanoid-derived volatile precursors are synthesized through tissue-specific transcriptional regulation of key biosynthetic enzymes in wampee fruit.

**Figure 5:**
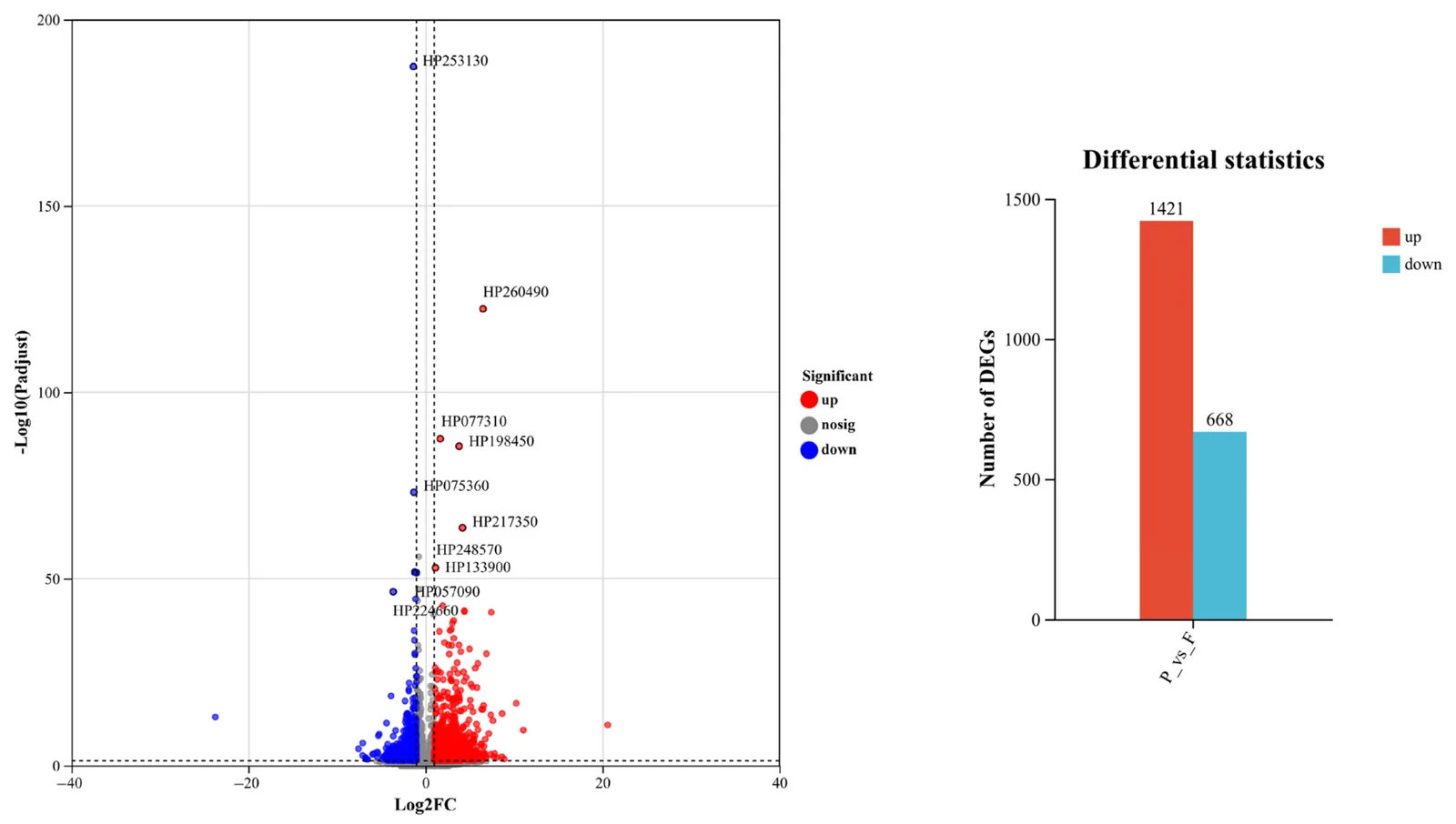
Volcano plot of differentially expressed genes (DEGs) between peel and flesh.

To globally visualize the overall pattern of transcriptomic variation between peel and flesh, all identified DEGs were displayed in a volcano plot (Figure 5), which exhibited obvious differences in gene expression magnitude and distribution between the two tissues.

### 3.4. Results of Integrated Metabolomic and Transcriptomic Analysis

**Figure 6:**
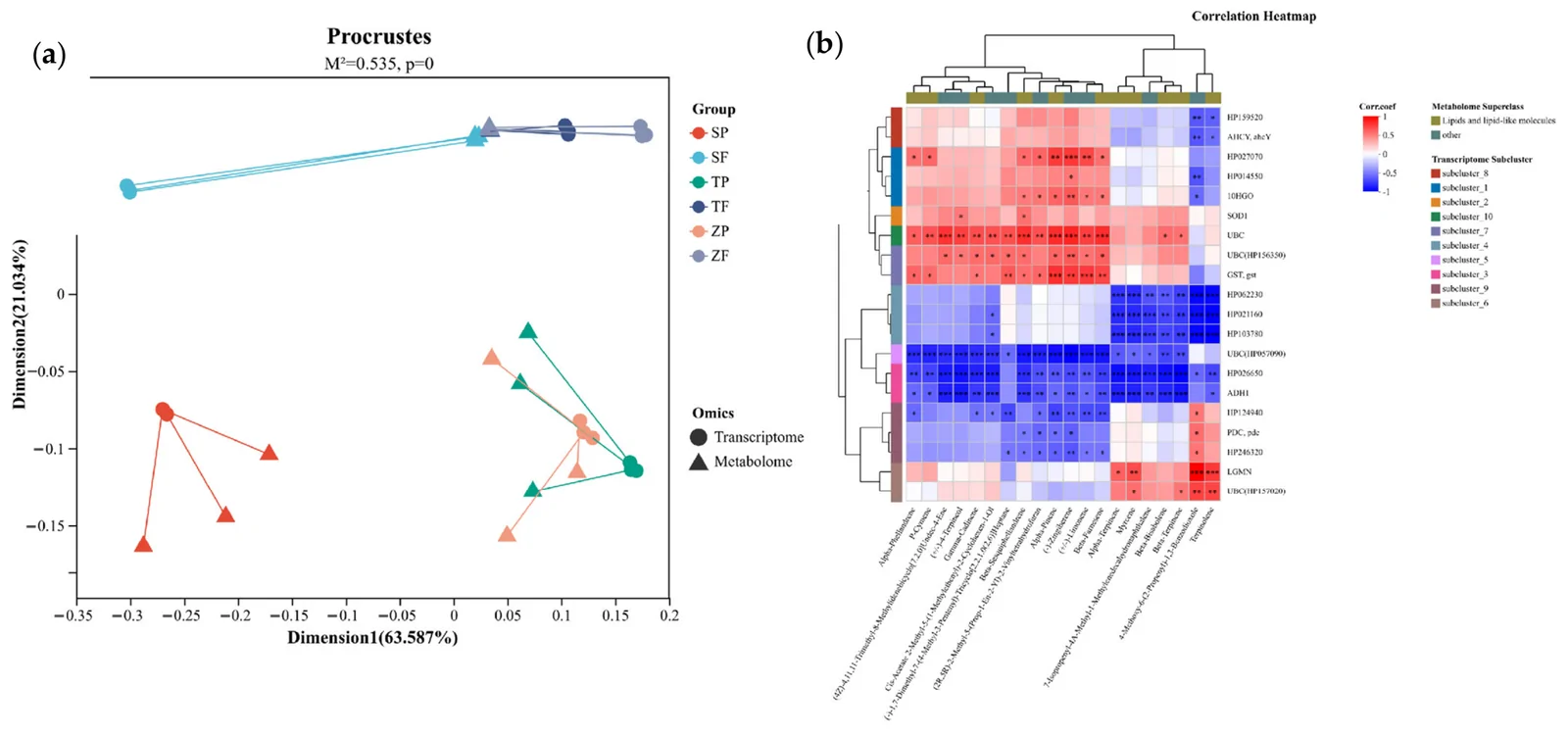
Integrated transcriptomic–metabolomic analysis revealing gene–metabolite associations in wampee fruit. (**a**) Procrustes analysis of transcriptome and metabolome datasets. Circles represent transcriptomic samples; triangles represent metabolomic samples. Lines connect corresponding samples from the same tissue–cultivar combination. M^2^ = 0.535, *p* < 0.001, indicating significant global concordance between omics layers. Dimension 1 (63.59%) separates peel (right) from flesh (left) samples. (**b**) Gene–metabolite correlation heatmap. Pearson correlation coefficients between 20 DEGs (rows) and top differential volatile metabolites (columns). Color scale: red, positive; blue, negative. Asterisks indicate significance levels (* *p* < 0.05, ** *p* < 0.01, *** *p* < 0.001). Row annotations denote transcriptome subclusters (1–10); column annotations indicate metabolome superclass categories.

To elucidate the molecular mechanisms underlying tissue-specific volatile accumulation, an integrated transcriptomic–metabolomic analysis was performed using Procrustes transformation and correlation network approaches. The Procrustes analysis revealed a significant concordance between the two omics datasets (M^2^ = 0.535, *p* < 0.001, Figure 6a), with Dimension 1 explaining 63.59% of the total variance and demonstrating that peel samples from all three cultivars consistently clustered toward the positive end of this axis for both transcriptome (circles) and metabolome (triangles) profiles, while flesh samples exhibited greater dispersion along Dimension 2 (21.03%). This congruence confirms that transcriptional reprogramming is tightly coupled with metabolic remodeling across tissues.

To further identify candidate regulatory genes driving volatile biosynthesis, a gene–metabolite correlation heatmap was constructed using Pearson correlation coefficients between 20 key DEGs and top differential volatile compounds (Figure 6b). The hierarchical clustering revealed three major transcriptional modules with distinct correlation signatures. Subcluster_8 (including SOD1, UBC, GST_gst, 10HGO) showed strong positive correlations (r > 0.5) with monoterpene-class metabolites such as α-phellandrene, γ-terpinene, p-cymene, and α-pinene, suggesting these genes may function as upstream regulators of terpenoid biosynthesis. Conversely, subcluster_4 (comprising ADH1, HP062230, HP021160, HP103780) displayed robust negative correlations (r < −0.7) with sesquiterpenes including β-bisabolene, myrcene, and β-elemene, indicating potential transcriptional repression of these pathways in flesh tissues. Notably, LGMN and HP246320 were uniquely associated with phenol ester derivatives, consistent with their enrichment in peel.

### 3.5. Results of qRT-PCR Gene Expression Validation

**Figure 7:**
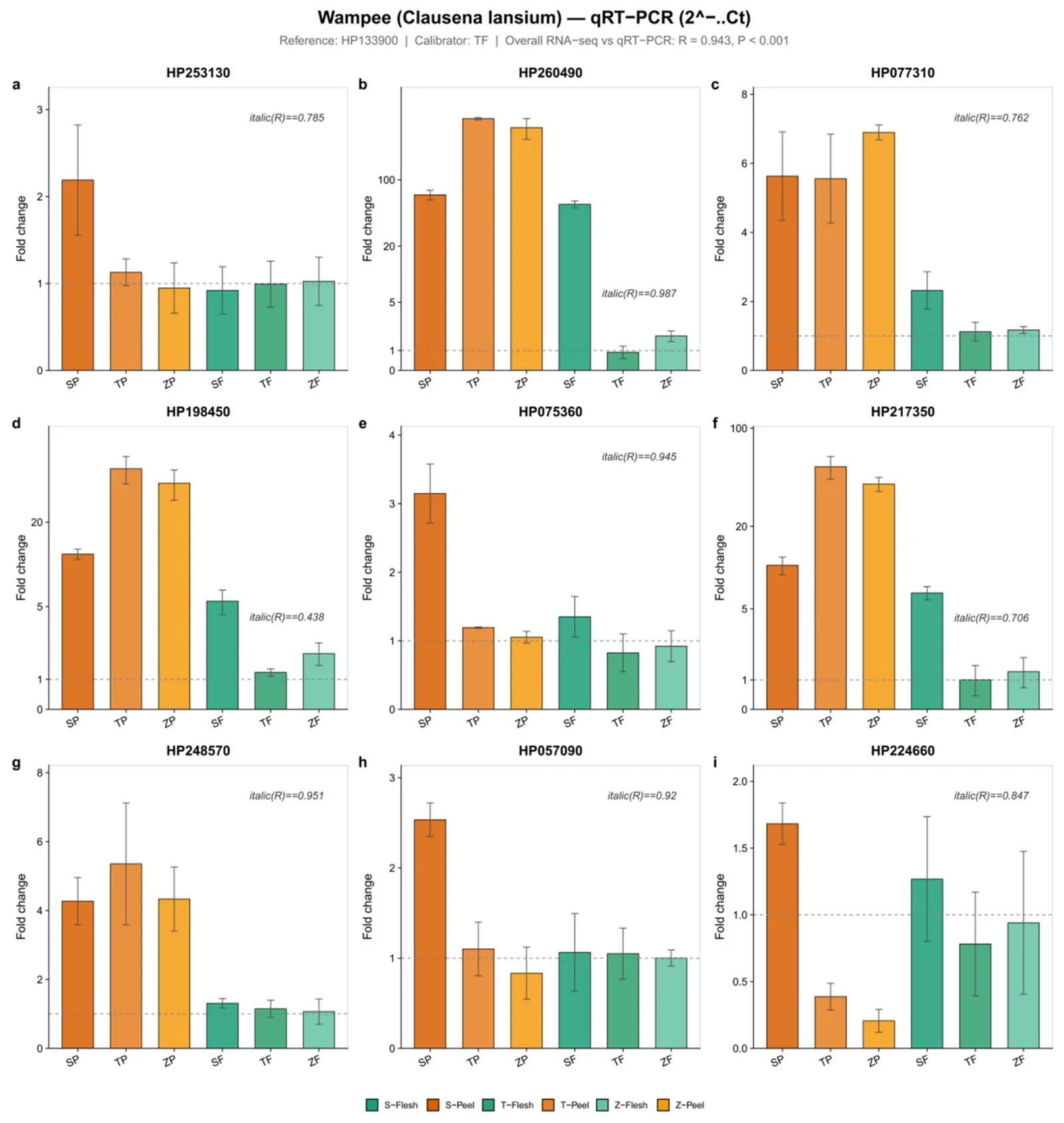
The qRT-PCR validation of differentially expressed genes involved in volatile biosynthesis. (**a**–**i**) Relative expression levels of 10 candidate genes measured by qRT-PCR (2^−ΔΔCt^ method). Samples are arranged as SP, TP, ZP (peel, orange shades), followed by SF, TF, ZF (flesh, teal shades). HP13390 (actin) served as the internal reference; TF was used as calibrator. Individual R values denote per-gene Pearson correlations between qRT-PCR and RNA-Seq data. Error bars indicate SD (*n* = 3).

**Figure 8:**
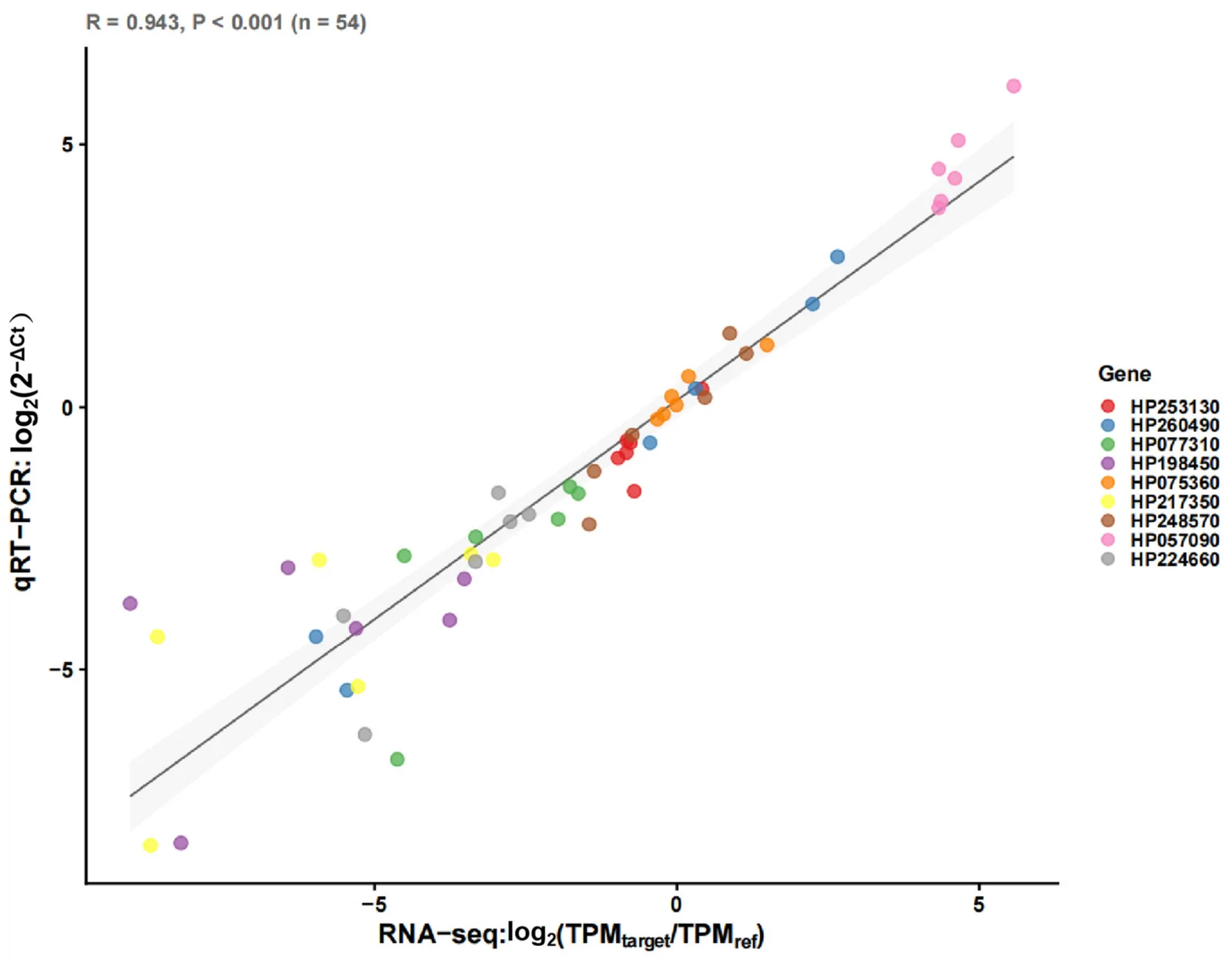
Correlation between RNA-Seq and qRT-PCR results. Scatter plot comparing gene expression values from RNA-Seq [log_2_(TPM_target/TPM_ref)] and qRT-PCR [log_2_(2^−ΔΔct^)]. R = 0.943, *p* < 0.001, *n* = 54. Each color represents one gene; gray shading indicates 95% confidence interval of the regression line.

To validate RNA-Seq reliability, 10 candidate DEGs were selected for qRT-PCR verification using HP13390 (actin) as the reference gene and TF as the calibrator. All genes exhibited consistent expression patterns between the two platforms (Figure 7a–i), with most showing higher abundance in peel tissues (orange bars) relative to flesh (teal). Notably, HP260490 displayed the strongest peel-enriched expression (up to ~150-fold), while HP198450 showed moderate variation among cultivars. Pearson correlation analysis confirmed a highly significant positive correlation between RNA-Seq (log_2_TPM) and qRT-PCR [log_2_(2^−ΔΔct^)] data across all 54 measurements (R = 0.943, *p* < 0.001, Figure 8), validating the accuracy of transcriptomic quantification.

## 4. Discussion

### 4.1. Tissue-Specific Volatile Profiling Reveals Distinct Metabolic Signatures Between Peel and Flesh

This study identified 288 metabolites across three wampee cultivars. The predominance of terpenoids (71.56–91.48% in peel; 61.88–67.22% in flesh) aligns with the established chemical phenotype of Rutaceae fruits [29,30,31]. In Citrus species, terpenoid proportions typically range from 40% to 70% [32,33]. The higher values observed here suggest that wampee may represent a particularly rich source of natural terpene extracts for food and cosmetic applications, although this remains to be validated through quantitative comparison with commercial Citrus cultivars.

A notable finding is the cultivar-specific enrichment of organoheterocyclic compounds in Shanyellowpi flesh (SF, 40.45%), a pattern not observed in the other two cultivars. Heterocyclic volatiles (pyrazines, furans, thiazoles) are typically associated with thermal processing (Maillard reaction) [34,35]. However, several studies have documented their natural occurrence in tropical fruits, where they contribute cooked, nutty, or roasted aroma notes [36,37]. The biochemical origin of this enrichment remains unclear. Amino acid catabolism or lipid oxidation pathways may generate furan precursors in a cultivar-specific manner [38,39], but this hypothesis requires targeted metabolite flux analysis to confirm.

In contrast to the terpenoid-dominant peel, wampee flesh exhibited higher accumulation of esters and alcohols. This pattern is consistent with the established biology of fleshy fruits, where alcohol acyltransferases (AATs) catalyze ester formation during ripening [40,41]. The “flesh > peel” accumulation trend observed here mirrors that reported in apple (*Malus domestica*), where *MdAAT1/2* expression peaks in fruit flesh [42,43]. Additionally, the exclusive enrichment of phenol esters in Heijingang peel (ZP, 1.34%) suggests a cultivar-specific metabolic signature potentially driven by BAHD acyltransferase activity [44,45]. Whether this enrichment correlates with specific sensory attributes (e.g., astringency, bitterness) remains to be established through GC–olfactometry. Beyond pome and Citrus fruits, tissue-specific partitioning of volatiles driven by alcohol acyltransferases and terpene synthases has been reported in banana, mango, strawberry, and grape, indicating that the peel-versus-flesh metabolic divergence observed here reflects a conserved strategy across diverse fruit crops.

### 4.2. Transcriptomic Insights: Phenylpropanoid and Terpenoid Pathways as Core Regulatory Hubs

Phenylpropanoid biosynthesis emerged as the most significantly enriched pathway among differentially expressed genes (DEGs) (Rich factor ≈ 0.92, Padj < 0.001), followed by monoterpene biosynthesis and sesquiterpenoid biosynthesis. This finding is biologically significant. The phenylpropanoid pathway supplies both volatile phenylpropanoids (e.g., methyl benzoate, phenylacetaldehyde) and phenolic antioxidants that modulate aroma stability [46,47]. In peach (*Prunus persica*), phenylpropanoid-derived volatiles are key aroma contributors. Their accumulation is transcriptionally regulated by MYB and bZIP transcription factors [48,49]. Whether similar regulators control phenylpropanoid-derived volatiles in wampee remains to be determined.

The co-enrichment of terpenoid biosynthesis pathways provides transcriptional evidence for the observed terpenoid dominance in volatile profiles. Terpene synthases (TPS) constitute a large multi-gene family in plants, with members showing distinct spatial and temporal expression patterns [50,51]. In Citrus, *TPS* are predominantly expressed in flavedo (outer peel), consistent with the tissue localization of oil glands [52,53]. The strong positive correlation between *TPS* expression (e.g., HP075360, HP217350) and monoterpene accumulation (r > 0.7, *p* < 0.01; Figure 6b) corroborates this spatial coupling. However, correlation does not establish causality. Functional validation (e.g., heterologous expression, enzyme assays) is needed to confirm the specific roles of these TPS genes in vivo.

Notably, oxidoreductase activities (including *SOD1*, *GST*, and *10HGO*) correlated with monoterpene levels (Subcluster_8, Figure 6b). *SOD1* and *GST* are involved in *ROS* homeostasis, which modulates *TPS* activity via redox signaling [54,55]. *10HGO* (10-hydroxygeraniol oxidase) is a key enzyme in iridoid biosynthesis, suggesting a potential role in terpene modification or downstream product formation [56,57]. These findings highlight the multifaceted regulation of terpenoid metabolism, extending beyond TPS genes to include stress-responsive and secondary metabolic enzymes. The relative contributions of these enzymes to terpenoid biosynthesis remain to be quantified.

### 4.3. Integrated Multi-Omics Analysis Reveals Candidate Regulators of Volatile Metabolic Divergence

Integration of transcriptomic and metabolomic data via Procrustes analysis revealed a significant global concordance between the two omics layers (M^2^ = 0.535, *p* < 0.001; Figure 6a). This confirms that transcriptional reprogramming is tightly coupled with metabolic remodeling across tissues. This congruence is consistent with previous multi-omics studies in Citrus [58,59] and apple [60,61], where gene–metabolite correlation networks have successfully identified candidate regulatory genes.

The gene-metabolite correlation heatmap (Figure 6b) revealed three major transcriptional modules with distinct correlation signatures. The positive correlation module (Subcluster_8) suggests that oxidative stress responses and secondary metabolic enzymes may act as co-regulators of terpenoid biosynthesis, potentially via substrate channeling or compartmentalization [62,63]. The negative correlation module (Subcluster_4, including ADH1) indicates potential competitive substrate utilization between alcohol fermentation and terpene biosynthesis. Alternatively, ripening-associated transcriptional programs may repress sesquiterpene biosynthesis in flesh tissues [64,65]. These interpretations are speculative and require experimental validation.

The unique association between *LGMN* (legumain, a cysteine protease) and phenol esters in Heijingang peel (ZP) is particularly intriguing. *LGMN* is involved in vacuolar protein processing and senescence [53,66]. Its specific correlation with phenol esters suggests a potential role in phenylpropanoid precursor mobilization or *BAHD* acyltransferase activation via proteolytic processing. This finding warrants further investigation using targeted gene expression assays and enzyme activity measurements. At present, the mechanistic link between *LGMN* and phenol ester accumulation remains correlative.

### 4.4. Comparative Perspectives, Limitations, and Future Directions

Compared to previous studies on wampee volatiles [29,54], our study provides a 4.6-fold increase in chemical coverage. This improvement is attributable to methodological advances (IL-based HS–GC–MS) and comprehensive tissue–cultivar sampling. The volatile profile of wampee exhibits both Rutaceae family terpenoid dominance and species-specific features (organoheterocyclic enrichment, phenol ester accumulation). These findings may have implications for flavor breeding and value-added product development (e.g., essential oil extraction) [55,56], although direct sensory validation is lacking.

Several limitations should be acknowledged. First, volatile quantification was performed using relative percentages rather than absolute concentrations. This precludes direct quantitative comparison with literature reports [57,58]. Future studies should incorporate stable isotope-labeled internal standards to enable absolute quantification. Second, transcriptomic analysis was conducted at a single developmental stage. This limits insights into temporal dynamics [59,60]. Longitudinal sampling across fruit development would elucidate stage-specific metabolic switches. Third, functional validation of candidate genes (e.g., *TPS*, *AAT*, *BAHD*) is lacking. Heterologous expression (e.g., in *E. coli* or yeast) and transient expression (agroinfiltration) assays are needed to confirm enzymatic activities [61,62].

Future research should prioritize: (1) genome-assisted identification of *TPS* and *AAT* gene families; (2) CRISPR/Cas9-mediated functional validation of candidate regulators; (3) GC–olfactometry to link volatile profiles with sensory perception [63,64]; and (4) QTL mapping or GWAS to identify genetic loci underlying volatile variation for marker-assisted breeding [65,66]. In conclusion, this study provides a comprehensive volatile and transcriptomic atlas of wampee fruit, revealing pronounced tissue-specific and cultivar-specific metabolic divergence. The integration of multi-omics data identifies candidate regulatory genes and metabolic pathways underlying volatile biosynthesis. These findings provide a foundation for future metabolic engineering and breeding efforts aimed at enhancing fruit quality in this understudied Rutaceae crop. These findings support our hypothesis that tissue-specific transcriptional regulation underlies volatile metabolic divergence in wampee. The precise contributions of individual candidate genes (e.g., *TPS*, *AAT*, *BAHD*, *GST*, *SOD1*, *10HGO*, *MYB*) remain to be established through targeted functional studies, such as heterologous expression (*E. coli* or yeast), transient expression (agroinfiltration), or virus-induced gene silencing (*VIGS*) assays, which are required to confirm enzymatic activities and regulatory roles.

## 5. Conclusions

This study provides the integrated volatile and transcriptomic atlas of wampee (*Clausena lansium*) pericarp and flesh across three cultivars, identifying 288 volatile metabolites. Terpenoids dominated the volatile profile in both tissues, accumulating at significantly higher levels in peel (71.56–91.48%) than in flesh (61.88–67.22%), whereas flesh preferentially accumulated esters and alcohols, reflecting tissue-specific metabolic partitioning. Integrated multi-omics analysis nominated candidate *TPS*, cytochrome *P450*, *MYB*, and oxidoreductase (*SOD1*, *GST*, *10HGO*) genes as potential regulators of tissue-specific volatile biosynthesis.

Transcriptomic profiling identified phenylpropanoid biosynthesis as the most significantly enriched pathway among DEGs, followed by monoterpene and sesquiterpenoid biosynthesis, providing a transcriptional basis for the observed terpenoid dominance. Collectively, these findings establish a foundation for molecular marker-assisted breeding and targeted flavor modulation in this understudied Rutaceae crop, while the precise contributions of individual candidate genes await functional validation.

## Figures and Tables

**Table 1:** Characteristics of the three wampee cultivars.

Cultivar Code	Cultivar Name
S	Shanyellowpi
Z	Heijingang
T	Bingtangxin

**Table 2:** Primer sequences used for qRT-PCR analysis.

Gene ID	Forward Primer (5′–3′)	Reverse Primer (5′–3′)	Product Length (bp)	Tm (°C)
HP253130	TGGAGTACGGTTCGGTGAGG	CTCATCTGCCATAATCCCAAG	180	60.2
HP260490	GCTAGTGTTGCTCGTTGGGG	AACAATAATGTTGTTGTGGCG	168	59.8
HP077310	GTTGTTGATAGTTGAACTGT	TCAATGTTCCGGAATCAAGAT	156	60.5
HP198450	GGAAGCTAAAATCAAGCAAAC	TCGTTCTTTGAACCATATTTC	145	59.9
HP075360	GGAATATTTGATTTTGTAGAA	CATCATCTTATCACATCCACA	162	60.1
HP217350	CCAAGTAACTACTTCCGAAAG	CGTGTAATATGGTGCAGAACT	148	60.3
HP248570	CAAAGCAGCCTGGTTTAGTGA	CACATCCACTTCAATCCAGAG	155	59.7
HP133900	GATGACGCTGGACTGGACGAA	TCAATGGCACTAATCCCAAGT	170	60.4
HP057090	GATGGTCCTACTAAGGTACAT	AATTTGGGTAAAACATTCCAG	140	59.6
HP224660	CAGCCAAGAGAGAGTTCAACC	TGTGTTGATCACCGTGGTGTT	158	60
Actin	CACTCTTCCAGCCTTCCTTC	GTACAGGTCTTTGCGGATGT	150	60

**Table 3:** Statistical Table of Compound Classification.

Volatile Class I	Number	SP (%)	SF (%)	TP (%)	TF (%)	ZP (%)	ZF (%)
Terpenoids	83	71.5592	44.8793	91.478	61.8812	90.0444	67.2226
Esters	45	0.2814	0.6174	2.0218	3.0707	1.0846	5.6644
Organoheterocyclic compounds	31	17.787	40.4508	1.1387	4.6683	0.2451	1.2275
Hydrocarbons	27	4.5808	9.5238	1.6792	8.4362	1.7139	6.2248
Alcohols	25	2.9078	2.649	0.9389	13.3616	1.689	8.736
Aldehydes	19	0.5708	0.3332	1.0201	2.3347	1.2849	4.1203
Ketones	16	0.6385	0.6524	0.6104	2.8594	0.4771	4.7645
Unknown	12	0.01	0.0368	0.0181	0.4665	0.0275	0.2217
Phenols	10	0.1531	0.2373	0.1922	0.5009	1.1248	0.6213
Phenol ethers	5	0.8741	0.2619	0.1933	0.8468	0.2489	0.0931
Phenol esters	4	0.0073	0.0032	0.0336	0.0088	1.3414	0.1616
Ethers	3	0.5813	0.0314	0.4079	0.1789	0.5405	0.2116
Organonitrogen compounds	3	0.0118	0.0524	0.1341	0.3386	0.0061	0.3939
Acids	2	0.0201	0.24	0.1154	0.8565	0.1611	0.157
Haloalkanes	2	0.0146	0.0122	0.0121	0.1041	0.0055	0.0425
Organosulfur compounds	1	0.0023	0.0189	0.0061	0.0868	0.0053	0.1369

## Data Availability

The raw data supporting the conclusions of this article will be made available by the authors on request.

## Supplementary Materials

The following supporting information can be downloaded at: https://www.mdpi.com/article/10.3390/metabo16080598/s1: Table S1: Overview Table of Metabolite Annotation Information; Table S2: Sequencing Data Statistics; Table S3: Sequencing Data Annotation Information.
